# Real-life lessons on omalizumab and food allergy: The potential for unexplored outcomes^☆^

**DOI:** 10.1016/j.waojou.2026.101390

**Published:** 2026-04-25

**Authors:** Lucia Lo Scalzo, Francesca Galletta, Arianna Cafarotti, Ulugbek Nurmatov, Ludger Klimek, Alessandro Fiocchi, Stefania Arasi

**Affiliations:** a Pediatric Allergology Unit, Bambino Gesù Children's Hospital (IRCCS), Rome, Italy; b Pediatric Unit, Department of Human Pathology of Adult and Childhood Gaetano Barresi, University of Messina, Messina, Italy; c Division of Population Medicine, School of Medicine, Cardiff University, Wales, UK; d Center for Rhinology and Allergology, Wiesbaden, Germany

**Keywords:** Biologics, anti-IgE, Omalizumab, IgE-mediated food allergy, Real-life studies

## Abstract

**Introduction:**

IgE-mediated food allergy (FA) represents an increasing global health burden, with rising prevalence and limited therapeutic options beyond allergen avoidance and emergency management. The approval of omalizumab as the first biologic to reduce reactions after accidental food exposure marks a turning point in FA care. While randomized controlled trials (RCTs) have demonstrated its efficacy in raising reaction thresholds and reducing allergic responses, real-world studies provide complementary evidence capturing outcomes relevant to everyday life.

**Methods:**

This review integrates evidence from sources of observational studies, including cohort, case series, and case reports evaluating omalizumab in patients with FA, including complex phenotypes and populations typically excluded from RCTs.

**Discussion:**

Real-world data demonstrate that omalizumab can prevent severe reactions in atypical and non-ingestive FA phenotypes, increase reaction thresholds as monotherapy, and enable dietary liberalization in a substantial proportion of patients. Observational cohorts consistently report improvements in food allergy–related quality of life, reduced anxiety, and enhanced confidence in food management. Omalizumab appears effective and well tolerated across complex populations, including patients with multiple comorbidities and very high total IgE levels, even beyond currently approved dosing limits. Emerging evidence also suggests that FA may require disease-specific dosing paradigms distinct from those used in asthma.

**Conclusions:**

Real-world evidence complements RCTs findings by highlighting the multidimensional benefits of omalizumab in FA, encompassing clinical protection, dietary freedom, and psychosocial well-being as well as dose fine-tuning and off-label scenarios. These data support its broader clinical value and inform future research, dosing strategies, and personalized application in routine care.

## Introduction

IgE-mediated food allergy (FA) is a growing global health concern, affecting up to 10% of children and a significant proportion of adults in industrialized countries, with a rising incidence of food-induced anaphylaxis reported worldwide.^1^^,^^2^ Despite major advances in diagnosis and prevention, the cornerstone of FA management remains strict allergen avoidance and emergency treatment with epinephrine, in case of accidental exposure.^3^^,^^4^ While these measures are life-saving, they impose a substantial burden on patients and families, severely impacting quality of life.^5^ The advent of biologic therapies has revolutionized the landscape of allergy care. In 2024, the U.S. Food and Drug Administration (FDA) approved omalizumab as the first medication to reduce allergic reactions after accidental food exposure, marking a paradigm shift in FA management.^6^ Evidence from randomized controlled trials (RCTs), including the pivotal OUtMATCH study, has demonstrated that omalizumab significantly increases reaction thresholds and reduces allergic reactions to multiple foods.^7^^,^^8^ Systematic reviews and meta-analyses further provide moderate-certainty evidence supporting its efficacy both as monotherapy and as an adjunct to oral immunotherapy (OIT) ^8^^,^^9^. However, RCTs, while essential for establishing efficacy and safety, are conducted under strictly controlled conditions and in highly selected populations. As a result, they often leave important clinical questions unanswered and fail to capture outcomes that are highly relevant in everyday practice. While RCTs primarily focus on controlled endpoints such as reaction thresholds and eliciting doses during oral food challenges (OFC), real-world studies provide insights into additional clinically meaningful outcomes, including improvements in food allergy–related quality of life (FA-QoL), dietary liberalization and reintroduction of previously avoided foods, reduced anxiety related to accidental exposures, protection against non-ingestive allergen exposure (eg, airborne exposure), and the management of atypical phenotypes such as food-dependent exercise-induced anaphylaxis (FDEIA).^8^

In this context, real-world evidence derived from case reports, case series, and large observational cohorts provides an indispensable complement to controlled trials. These studies offer valuable insights into patient groups underrepresented in RCTs, such as those with multiple comorbidities or very high IgE levels, and provide a more realistic understanding of treatment outcomes, safety, and clinical management in everyday settings.^10^

This narrative review aims to synthesize the lessons learned from real-life studies on omalizumab in FA. By critically analyzing clinical experiences across descriptive and analytical real-world observational evidence studies, including cohort, case series and case reports, it highlights clinically relevant outcomes that are not or not entirely captured by RCTs, identifies practical challenges, and outlines perspectives for future research and clinical application.

## Methods

### Information sources and search strategy

The international electronic databases EMBASE, Medline (Via Pubmed) as well as the search engine Google Scholar were searched to identify relevant articles in this field of research, published between 2006 and 2026. The search strategy included combinations of the following keywords and related terms: “omalizumab*”* AND (“food allergy” OR “IgE-mediated food allergy”) AND (“observational stud∗”, “cohort”, “case series”, “case report∗”, “real-world stud∗”). Reference lists of all included articles were also screened to identify additional relevant publications. Articles reporting incomplete or insufficient data (eg, conference abstracts or editorials) were excluded. The literature search and study selection were performed by 2 authors.

### Study eligibility criteria

Studies were eligible for inclusion if they were original observational studies, including quantitative, qualitative, descriptive, or analytical designs, investigating the role of omalizumab in the management of IgE-mediated food allergy. Eligible study designs included cohort studies, case series, and case reports. No restrictions were applied regarding patient age, number of food allergies, or presence of allergic comorbidities. Randomized controlled trials, systematic reviews, meta-analyses, and conference reports were excluded.

### Study records

#### Screening

Following removal of duplicates and application of search limits, 2 authors (LLS and FG) screened all titles and abstracts of citations and determined the potential for inclusion in the review. Full texts of relevant citations were obtained and read to determine eligibility, and data detailing the study characteristics and outcomes were extracted if the eligibility criteria were met.

#### Data extraction

For each included study, data were extracted on study aims, population, methods, analyses and key findings.

#### Quality appraisal and analysis

Given the narrative nature of this review and the heterogeneity of the included observational study designs, a formal risk-of-bias assessment was not performed.

The results of included studies were synthesized narratively (Table 1).

**Table 1:** Summary of observational and real-world studies evaluating omalizumab in patients with IgE-mediated food allergy.

Study	Study design	Population	Indication for omalizumab	Dosing strategy	Main outcomes	Follow-up
Arasi et al., 2024	Observational study	65 children with severe FA	Monotherapy (for asthma with assessment of FA threshold)	Standard dosing based on body weight and IgE levels	Increased reaction threshold, food reintroduction, improved ACT and FA-QoL	12-month follow-up with OFCs at 4-month intervals (T1, T2, T3)
Arasi et al.,2026	Prospective real-life observational study	76 children (6–18 years) with severe allergic asthma and severe FA (mostly multi-FA)	Monotherapy (for asthma with assessment of FA threshold)	Standard dosing based on body weight and IgE levels	Dose-dependent association with food desensitization; 61.5% achieved complete free diet and 26.2% partial food reintroduction	12 months (cohort follow-up up to 6 years)
Azzano et al., 2021	Multicenter observational cohort study	181 patients with FA undergoing multifood OIT	Adjunct to OIT	Variable dosing; analysis of dose–response (weight-based vs weight + IgE)	Higher OMA dose per weight associated with improved tolerance during initial food escalation and successful OIT progression	Follow-up during OIT and after OMA discontinuation; continued consumption assessed up to 2 years
Crisafulli et al., 2019	Case series	8 children with severe allergic disease; 5 with CMA, 6 with severe allergic asthma	Severe uncontrolled allergic asthma and/or adjunct to OIT for FA	Standard dosing based on body weight and IgE levels	Improved asthma control and facilitation of food desensitization	Up to 24 months
Fiocchi et al., 2019	Real-life observational study	15 children with severe asthma and concomitant FA	Monotherapy (for asthma with assessment of FA threshold)	Standard dosing based on body weight and IgE levels	Significant increase in food tolerance thresholds (≈8.6-fold); 70% of foods fully tolerated; marked reduction in accidental reactions	4 months
Mohamed et al., 2024	Case report	20-year-old woman with FDEIA	Prevention of FDEIA reactions	Omalizumab 300 mg subcutaneously every 4 weeks (off-label)	Resolution of exercise-induced reactions	3 months
Rafi et al., 2010	Prospective pilot study	22 patients with asthma and FA	Monotherapy (for asthma with assessment of FA)	Standard dosing based on body weight and IgE levels	Reduction or absence of food-allergic symptoms during accidental or deliberate re-exposure to sensitized foods	≥1 year treatment follow-up
Rocha et al., 2011	Case report	2 patients with multiple FAs and EoE	Severe FA with restrictive diet	300 mg every 2 weeks	Improvement of allergic symptoms and quality of life; no histologic improvement of EoE	6–10 months
Yee et al., 2019	Long-term follow-up study	37 patients with peanut allergy previously enrolled in OMA-facilitated OIT	Adjunct to OIT	Short-term OMA pre-treatment (∼12 weeks) before and during initial OIT phase	Rapid desensitization during OIT; long-term peanut consumption maintained in ∼51% of patients	Up to 72 months

**List of abbreviations: ACT**: Asthma Control Test; **CMA**: Cow's Milk Allergy; **EoE**: Eosinophilic Esophagitis; **FA**: IgE-mediated Food Allergy; **FA-QoL**: Food Allergy–related Quality of Life; **FDEIA**: Food-Dependent Exercise-Induced Anaphylaxis; **IgE**: Immunoglobulin E; **OFC**: Oral Food Challenge; **OIT**: Oral Immunotherapy; **OMA**: Omalizumab

## Real-world insights and clinical implication

### Lesson 1: Omalizumab may prevent severe reactions in non-ingestive and atypical food allergy phenotypes

Early case reports provided the first indication that omalizumab can prevent severe food-allergic reactions beyond classical ingestion-triggered exposures. These observations are particularly informative because they explore rare or atypical phenotypes that are systematically excluded from RCTs, by design, thereby filling an important real-world evidence gap.

One of the earliest descriptions involved a 16-year-old girl with anaphylaxis triggered by airborne chicken vapors, a setting in which allergen avoidance is often impractical.^11^ Omalizumab therapy (300 mg monthly) led to complete resolution of symptoms in daily life and, notably, enabled full tolerance during a subsequent OFC. This case highlights omalizumab's ability to protect against non-ingestive allergen exposure, expanding its clinical relevance to scenarios where environmental control is limited.

Similarly, case reports documented omalizumab's effectiveness in FDEIA. Mohamed et al^12^ described a young adult with wheat-triggered FDEIA who achieved complete symptom control on omalizumab, despite persistent reactions under standard preventive measures. These findings suggest that IgE-mediated pathways remain central even in cofactor-dependent phenotypes and that targeted IgE blockade may stabilize these complex clinical presentations.

Collectively, these reports demonstrate that omalizumab can provide clinically meaningful protection in scenarios where conventional avoidance strategies fail, underscoring its potential role in rare and high-risk clinical settings.

### Lesson 2: Omalizumab monotherapy increases reaction thresholds and enables dietary liberalization

Larger observational studies provided compelling evidence that omalizumab alone (ie, without concomitant OIT) can meaningfully increase food reaction thresholds.

Fiocchi et al^13^ reported that, after 4 months of omalizumab therapy, all of the 15 enrolled children with severe asthma and immediate reactions to ≥2 foods at the entry OFCs showed an increased eliciting threshold. Specifically, 9 children tolerated full serving sizes of all offending foods, 2 achieved complete tolerance to at least 1 food, and 4 showed partial improvement. Among those who reached full tolerance, previously avoided foods were successfully reintroduced into the diet.

Building on these results, a larger real-world study (OSAFA, Omalizumab in Severe Asthma and Food Allergy) by Arasi et al^10^ involved 65 patients with severe asthma and concomitant FA, and furtherly confirmed and extended these findings. In the first year of omalizumab therapy, 95/147 OFCs were negative allowing liberalization of 95 foods across 55 patients, while an additional 10 patients were able to introduce 12 foods in trace amounts. Remarkably, over 60% of participants achieved a free diet. However, interpretation of these findings should consider the heterogeneity of OFC protocols and definitions of tolerance across observational studies.

Of note, differently to current FDA indications in the United States, in the context of the Italian OSAFA cohort, all patients continued to assume the culprit food and to carry epinephrine on, according to the threshold of reactivity at the exit OFCs. None of the recruited patients stopped omalizumab; all of them have been informed about the role of co-factors in eliciting allergic reactions, including the half-life of omalizumab and the possible loss of the results achieved upon drug withdrawal.^10^

### Lesson 3: Omalizumab improves food allergy-related quality of life

Beyond immunological efficacy, real-world evidence highlights patient-centered benefits of omalizumab that are highly relevant to daily life. Consistent improvements in FA-QoL have been documented, alongside reductions in anxiety surrounding accidental exposures, and the restoration of dietary normality, as well as a marked decrease in allergic reactions (even upon *ad libitum* diet).

**Fig. 1:**
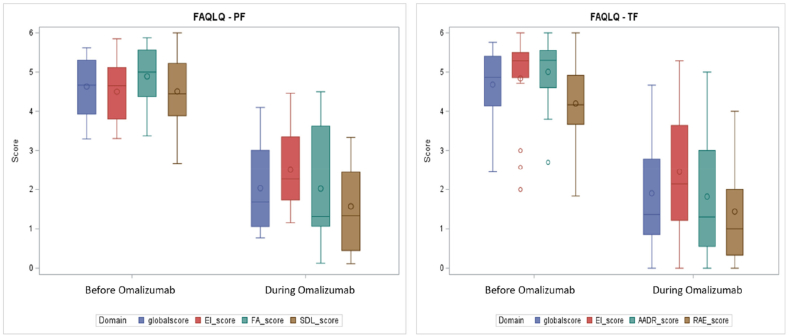
Box and whisker plots showing score of FAQLQ-PF and FAQLQ-TF before and during Omalizumab treatment in the OSAFA cohort. Reproduced from Arasi et al.^9^

Studies such as those by Arasi et al^10^ and Yee et al^14^ have shown that even treatment courses can substantial psychosocial improvements, with both children and their parents reporting enhanced emotional well-being and greater confidence in food management. Such outcomes, rarely quantified in RCTs, reflect dimensions of treatment success that matter most to patients and families (Fig. 1).

Taken together, these findings position omalizumab not merely as a desensitizing agent, but as a multidimensional therapy capable of meaningfully improving the lived experience of food-allergic individuals. By addressing both safety and psychosocial functioning, real-world data underscore omalizumab's broader clinical value and help bridge the gap between controlled trial efficacy and everyday clinical practice.

### Lesson 4: Omalizumab is effective and safe across complex patient populations

Real-world evidence consistently indicates the effectiveness and safety of omalizumab across complex patient populations with FA, including those with multiple, severe, or comorbid allergic conditions, that are typically underrepresented or excluded from RCTs.

One of the earliest prospective observations, by Rafi et al,^15^ evaluated 22 patients aged 4–66 years with persistent asthma and concomitant FA. Treatment with omalizumab according to asthma indication was associated with clinical improvement in all subjects, including reduced food-induced asthma, dermatitis, and anaphylaxis, with several patients tolerating previously reactive foods such as milk, egg, peanuts, and shellfish. No adverse events directly related to omalizumab were reported, providing preliminary evidence suggesting both efficacy and safety in the management of FA.

Another early case series, published by Rocha et al^16^ included 2 patients with multiple FAs and concomitant eosinophilic esophagitis who were treated with omalizumab to alleviate severe dietary restrictions and improve food tolerance. Both patients showed improvement in allergic symptoms, including tolerance to previously reactive foods and a reduction in anaphylactic reactions, along with better asthma and skin symptom control. However, endoscopic and histologic findings of eosinophilic esophagitis remained unchanged, suggesting that the esophageal inflammation in this subset may not be primarily IgE-driven.

Moreover, Crisafulli et al^17^ reported 8 children with severe allergic asthma and concomitant FA, predominantly to cow's milk and egg, treated with omalizumab for up to 2 years. All patients achieved improvement in asthma control, while most appeared to have increased food tolerance thresholds and a reduced frequency of accidental reactions; notably, 2 children successfully underwent milk OIT during treatment, and no serious adverse events were observed throughout follow-up.

These findings are consistent with results from larger observational cohorts, including the OSAFA studies, which suggest increases in tolerated doses across a range of allergens, safe reintroduction of previously excluded foods, and substantial improvements in asthma control in children with severe asthma and FA.^10^^,^^13^ Of note, considering omalizumab agnostic immunological mechanism (ie, not antigen specific), patients with allergy to multiple food and concomitant allergic comorbidities (eg, asthma, urticaria, nasal polyps, but not eosinophilic esophagitis nor eczema) may represent suitable candidates for treatment. Real-world evidence also suggests that individuals with a history of severe reactions or recurrent anaphylaxis despite appropriate avoidance strategies may derive substantial benefit from the protective increase in reaction thresholds observed with omalizumab. In addition, patients experiencing substantial FA-QoL impairment due to strict dietary restrictions or fear of accidental exposure may represent another group in whom treatment could be prioritized in clinical practice.

### Lesson 5: Omalizumab is well tolerated in patients with very high total IgE levels

Later series confirmed efficacy and mainly safety even in patients with extremely elevated IgE levels and off-label high-dose regimens. Omalizumab use is formally restricted to patients with total IgE ≤1850 U/L at baseline (updated in February 2024, previously 1500 kU/L in the leaflet),^18^ as recently reported, due to theoretical concerns about immune complex formation and renal risk. However, when omalizumab was used in patients with asthma and concomitant FA and basal IgE >1850 IU/mL, the safety profile has been shown to be optimal. Dinardo et al^19^ reported a two-year case series including 7 asthmatic children with multiple FAs, and treated with omalizumab (600–1200 mg monthly) and of note high serum IgE levels (mean serum tIgE level before treatment was 2158 [Min 1699–Max 3307] kU/L). All patients achieved full asthma control and reached complete desensitization. No adverse events were reported, confirming both efficacy and safety even beyond approved IgE dosing limits. These findings are particularly relevant, as such patients are typically excluded from RCTs.

### Lesson 6: Food allergy may require a distinct omalizumab dosing paradigm

In FA, optimal dosing of omalizumab is currently unclear. Omalizumab dosing typically follows the established guidelines for allergic asthma, which are based on the patient's body weight and baseline total IgE levels.

**Fig. 2:**
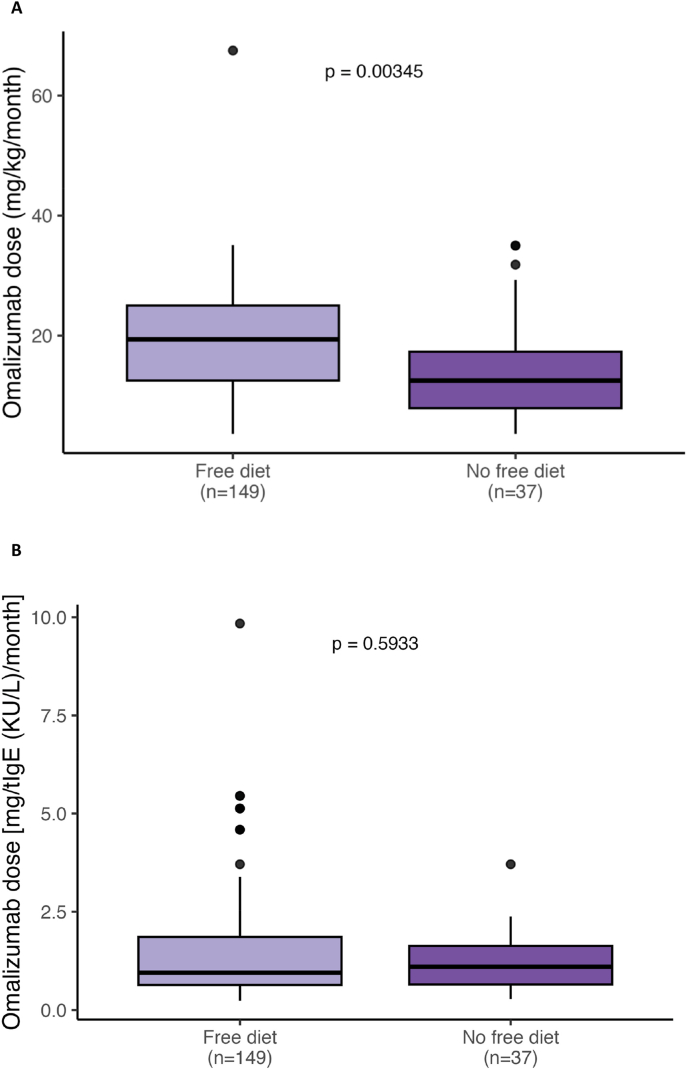
**Omalizumab Dose-Related Efficacy in a Cohort of Children With Severe Food Allergy.** Results from the OSAFA cohort. **2A.** Dose of Omalizumab (mg/kg/month) administered in all patients who have achieved at 12 month of treatment a free diet (n = 149) versus those who have not achieved a free diet (n = 37). The box represents the interquartile range (25th–75th percentile), the horizontal line indicates the median, whiskers extend to 1.5 × IQR, and outliers are plotted individually. **2B.** Dose of Omalizumab [mg/tIgE (KU/L)/month] administered in all patients who have achieved at 12 month of treatment a free diet (n = 149) versus those who have not achieved a free diet (n = 37). The box represents the interquartile range (25th–75th percentile), the horizontal line indicates the median, whiskers extend to 1.5 × IQR, and outliers are plotted individually. Reproduced from Arasi S.^21^

Observational research has begun to challenge asthma-derived dosing paradigms for omalizumab in FA. In a large Canadian cohort (n = 181) of FA children and young adults undergoing omalizumab preliminarily and during multifood OIT, Azzano et al^20^ demonstrated that the dose-dependent effects of omalizumab on reactivity thresholds was best predicted using body weight alone, independently of total IgE levels prior to omalizumab commencement. Similar results have been recently shown with omalizumab as monotherapy in the OSAFA cohort (Fig. 2).^21^ These findings suggest that FA may require disease-specific dosing strategies, independently from serum total IgE in serum. Furthermore, the Canadian study also suggests that the specific activity (ie, the ratio of allergen-specific to total IgE may help predict reaction risk during dose tapering, providing a potential biomarker for individualized treatment planning.^20^

## Conclusions

Accumulating real-world evidence indicates that omalizumab represents a significant advance in the management of FA, extending beyond the efficacy demonstrated in RCTs. Observational studies and clinical experience consistently show that omalizumab can increase reaction thresholds, reduce the frequency and severity of allergic reactions, and enable meaningful dietary liberalization, even in patients with complex phenotype (such as those with multiple FAs, severe reactions or recurrent anaphylaxis notwithstanding a proper avoidance behavior), multiple comorbidities, or very high total IgE levels who are typically excluded from RCTs, with a significant improvement also in FA-related quality of life.

Furthermore, emerging evidence suggests that FA may require disease-specific omalizumab dosing strategies distinct from asthma-based paradigms, underscoring the need for tailored approaches and further investigation. While current findings support the safety and effectiveness of omalizumab across diverse and high-risk populations, key questions remain regarding optimal dosing, treatment duration, timing and modality of discontinuation tapering strategies, and persistence of tolerance after withdrawal.

Although encouraging, these findings derive largely from heterogeneous observational studies and should therefore be interpreted with appropriate caution, considering potential selection bias, variable follow-up, and heterogeneity in outcome definitions (eg, reaction thresholds, dietary liberalization)**.** Standardized definitions for outcomes would further improve comparability across studies and facilitate translation into clinical practice and guidelines.

## Abbreviations

FA: IgE-mediated food allergy; FA-QoL: Food Allergy-related Quality of Life; FDA: Food and Drug Administration; OFC: Oral food challenge; OIT: Oral immunotherapy; RCTs: Randomized controlled trials; FDEIA: Food-dependent exercise-induced anaphylaxis.

## Ethics statement

The study was considered exempt from ethics because of its nature.

## Author contributions

SA and LLS conceived this review. LLS and FG undertook searches, data extractions, critical appraisal. The study was drafted by LLS, FG and SA; then the manuscript was revised by all co-authors.

## Authors’ consent for publication

Yes (from all authors).

## Disclosure of the use of generative AI and AI-assisted technologies

Nothing to disclose.

## Funding

This work was supported by “TAHyTi (Thresholds Allergen Hypoallergenic Therapeutic): Prospective evaluation of reactivity Thresholds for Allergens and HYpoallergenic food for potential Therapeutic applications (Project no. PNRR-MCNT2-2023-12377575). This work was supported also by the Italian Ministry of Health with “Current Research funds”.

## Declaration of competing interest

S. Arasi declares that she has participated as an advisory board member, and/or consultant, and/or speaker/chair at scientific meetings for Aimmune, DBV, Ferrero, Mabylon, Novartis, Stallergenes Greer, Thermo Fisher Scientific and Ulrich outside the submitted work. Funded research (Institution) from Italian Minister of Health and Italian Minister of Education.

A. Fiocchi has received Speaker honoraria and advisory panel consultancy outside the submitted work for Nutricia, Abbott, Danone, Stallergenes, DBV, Novartis. Funded research (Institution) from Sanofi, Novartis, Ferrero, DBV, GSK, Astrazeneca, Hipp GmBDH, Humana SpA. IJA reports personal fees from Bayer, Bial, Cipla, Eurodrug, Faes Farma, Gebro, Glenmark, Opella, Menarini, MSD, Roxall and Sanofi outside the submitted work.

L. Klimek has received research grants from Allergy Therapeutics/Bencard, Great Britain/Germany; ALK-Abelló, Denmark; Allergopharma, Germany; Aimmune, USA; ASIT Biotech, Belgium; AstraZeneca, Sweden; Bionorica, Germany; Biomay, Austria; Boehringer Ingelheim, Germany, Circassia, USA; Chiesi, Italy; Cytos, Switzerland; Curalogic, Denmark; HAL, Netherlands; Lofarma, Italy; Menarini, Italy; Viatris/Mylan, USA; Novartis, Switzerland; Leti, Spain; ROXALL, Germany; GlaxoSmithKline (GSK), Great Britain; Sanofi, France; Stallergenes, France; Thermofisher, USA; and/or has served on the speaker's bureau or was consulting for the above-mentioned pharmaceutical companies. LK is the current President of German Society of Allergology AeDA, Vice-President of the European Academy for Allergy and Clinical Immunology (EAACI), Vice-President of the German Academy for Allergy and Environmental Medicine, and Editor-in-Chief of AllergoJournal and AllergoJournal International. All are outside of the submitted work.

All other authors have no conflict of interest within the scope of the submitted work.
